# Transcriptome Analysis of Testes and Uterus: Reproductive Dysfunction Induced by *Toxoplasma gondii* in Mice

**DOI:** 10.3390/microorganisms8081136

**Published:** 2020-07-28

**Authors:** Junjie Wang, Tanghui Liu, Yasser S. Mahmmod, Zipeng Yang, Jiexing Tan, Zhaowen Ren, Xirui Zhang, Xiaoying Yang, Xiu-Xiang Zhang, Zi-Guo Yuan

**Affiliations:** 1 College of Veterinary Medicine, South China Agricultural University, Guangzhou 510642, Guangdong, China; dgwj2j@163.com (J.W.); 15622138776@163.com (T.L.); yzp19960924@163.com (Z.Y.); tanjiexing24@163.com (J.T.); rzw19940215@outlook.com (Z.R.); sivnmarello@gmail.com (X.Z.); 20183073090@scau.edu.cn (X.Y.); 2 Key Laboratory of Zoonosis Prevention and Control of Guangdong Province, Guangzhou 510642, Guangdong, China; 3 Key Laboratory of Zoonosis of Ministry of Agriculture and Rural Affairs, South China Agricultural University, Guangzhou 510642, Guangdong, China; 4 Guangdong Laboratory for Lingnan Modern Agriculture, Guangzhou 510642, Guangdong, China; yasserpcr@gmail.com; 5 Infectious Diseases, Department of Animal Medicine, Faculty of Veterinary Medicine, Zagazig University, Zagazig 44511, Sharkia, Egypt; 6 Veterinary Sciences Division, Al Ain Men’s College, Higher Colleges of Technology, Al Ain 17155, Abu Dhabi, UAE; 7 College of Agriculture, South China Agricultural University, Guangzhou 510642, Guangdong, China

**Keywords:** *T. gondii*, testes, uterus, transcriptome analysis, RNA-Seq, DEGs

## Abstract

*Toxoplasma gondii* (*T. gondii*) infection in female mammals during pregnancy can result in poor pregnancy. Similarly, it can result in male reproductive disorders in male mammals. Although the testes and uterus have very different biological makeup, they are still both attacked by *T. gondii* resulting in reproductive dysfunctions. We hypothesized that there are significant common genes in the testes and uterus that interact with *T. gondii*. Finding out and studying these genes is vital to understand the infection mechanism of *T. gondii* and the induced disease pathogenesis. To achieve this goal, we built a mice model of acute infection with *T. gondii* and the testes and uterus of the mice were sequenced by RNA-Seq. A total of 291 and 679 significantly differently expressed genes (DEGs) were obtained from the testes and the uterus, respectively. In the Gene Ontology (GO) analysis, part of the DEGs in the testes and uterus were related to 35 GO functions. When compared with the KEGG database, seven pathways affecting both the testes and uterus during the course of *T. gondii* infection were identified. In addition, Toxoplasmosis can significantly affect the expression of *Nlrp5* and Insc leading to negative outcomes in the host. On the other hand, the host regulates *Gbp7*, *Gbp2b*, and *Ifit3* to defend against *T. gondii* infection.

## 1. Introduction

*Toxoplasma gondii* (*T. gondii*) is a “clever” obligate intracellular protozoan parasite, affecting most warm-blooded mammals [1]. In fact, about one third of the world population has been infected by *T. gondii* [2]. The large number of infections has led researchers to study the “symbiotic” relationship between *T. gondii* and the different hosts [3]. However, as an exogenous parasite, *T. gondii* is always harmful to the body organs, where infection commonly occurs via oral, blood, and placental routes [4]. *T. gondii* infection in pregnant female animals may lead to fetal malformations, stillbirths, and abortions [5]. Meanwhile, in male animals, it can cause reproductive system damage, sexual dysfunction, and fertility disorders [6]. Previous researchers have used epidemiological, pathological, and serological approaches to detect the DNA of *T. gondii* in the semen of infertile patients and they observed significant cellular damage in the testicular tissue [7,8,9], proving that there is an exact association between *T. gondii* and male infertility.

Anatomically, there are many seminiferous tubules in the testis, which are the places of spermatogenesis. The structure of these tubules contains spermatogonia cells, spermatogenic cells, and sperms from outside to inside. The seminiferous tubules are interspersed with the supporting cells, and the junctional complexes (mainly tight junctions), which are located between the supporting cells resulting in the formation of the blood–testis barrier (BTB). The BTB has many functions, for instance it restricts the substances in plasma from entering the seminiferous tubules, prevents the sperms from entering the blood, and forms the immune exemption zone as well as it provides a stable environment for the orderly occurrence of sperm [10,11]. The placenta is responsible for the material exchange between the mother and the fetus during pregnancy [12], which, in turn, depends on the multilayers structure of the placenta. Structurally, the placenta is composed of syncytiotrophoblast, the villous endothelial cells, and the thin interstitium, which together form the placental barrier that regulates the material exchange rate and selectivity. Additionally, it ensures that the maternal villous space and fetal circulation are separated [13]. *T. gondii* traverses the physical barrier via lymphatics and blood vessels to reach the organs of the host. Both males and females have specific physiological barriers. *T. gondii* go through BTB when they invade the testes, and as they pass from the female to the fetus by vertical transmission, they need to pass the placental barrier.

*T. gondii* infection in pregnant female mammals can result in poor pregnancy. The majority of previous research focused on the relationship between *T. gondii* and pregnant animals, but only few of them considered *T. gondii* infection in male mammals despite the evidence that *T. gondii* also invades the testes, causing male reproductive disorders. Although BTB and the placental barrier are different in their anatomical structures, they have a similar function through acting as a physiological barrier for the isolation of tissue and blood. However, *T. gondii* in the blood can penetrate BTB or the placental barrier into tissues. In our previous work, after comparing the differential expression of brain proteins in rats and mice after *T. gondii* infection, we found that effective proteins can affect the invasion of the blood–brain barrier by *T. gondii* [14]. This study deepened our thinking about *T. gondii* breaking through the biological barrier and shed the light on new important queries. For instance, do BTB and placental barrier have common key molecules, which facilitate *T. gondii* invasion to the reproductive system? Keeping that in mind, we carried out this research to understand the mechanism of *T. gondii* invasion to reproductive organs.

Omics technologies including transcriptomics, proteomics, and metabolomics become important technologies in the current scientific research [15]. Transcriptomics is one of the high-throughput technologies that can identify the type and copy-number changes of mRNA when the cell is functional [16]. Genetic studies have shown that mRNA acts as a “bridge” for the transmission of genetic information between DNA and proteins. Therefore, it poses a valuable source for identifying the expression of all genes in a cell with a specific time and space. Using the omics technologies, we sequenced number samples from the testes of male mice and the uterus of pregnant mice, and then, we performed a transcriptomic analysis, and we found that there are significant common genes in the testes and uterus that interact with *T. gondii*. Trying to find and identify the key genes of *T. gondii* invasion into the reproductive system is vital to understand the infection mechanism of *T. gondii* and the induced disease pathogenesis.

## 2. Materials and Methods 

### 2.1. Animals and Experiment Set-Up

Eight-weeks old specific-pathogen free (SPF) Kunming (KM) mice, purchased from Guangdong Medical Laboratory Animal Center, were used in this study. Male mice were randomly divided into two groups (*n* = 5), including infected and control groups. Two female mice were mated with one male mouse. Female mice were judged as the 0th day of pregnancy when vaginal plug occurred. Then, the pregnant mice were randomly divided into two groups (*n* = 5), including infected and control groups. On the 7th day of female mice pregnancy, they were infected with *T. gondii*. Two mice groups were injected intraperitoneally with 0.2 mL of physiological saline containing 1 × 10^2^ *T. gondii* tachyzoite RH strain (type I) and labelled as infected groups. In our previous work, we have previously studied the optimal number of RH strain infections per mouse, in order to minimize damage by *T. gondii* infection [17]. Meanwhile, the control group was injected with an equal dose of physiological saline. On the 12th day of pregnancy in female mice (5th day of *T. gondii* infection in infected group), all the mice were euthanized by CO_2_. The mouse testes and uterus were quickly harvested and collected for subsequent experiments. One sample was taken from one mouse, and the number of samples was equal to the number of mice. The samples from infected groups were named by gender into MaleTox and FemaleTox groups, and the samples from control groups were also divided into MaleCtrl and FemaleCtrl groups according to gender. Three sets of biological replicates were implemented, for a total of 60 KM mice (male: female = 1:1).

Tachyzoites of the highly virulent RH strain of *T. gondii* were preserved in our laboratory (Laboratory of Parasitology, College of Veterinary Medicine, South China Agricultural University) and maintained by serial intraperitoneal passage in KM mice.

### 2.2. Evaluation of Sperm Quality Parameters

The epididymis was isolated from the reproductive system of 30 male mice. Afterward, it was cut into small pieces, then 1 mL PBS was added and was placed in a CO_2_ incubator at 37 °C for 15 min to make a sperm suspension. Part of the sperm suspension was removed and the total number of spermatozoa was calculated using a hemocytometer. In addition, the premixes of 5% eosin and 10% nigrosine were mixed with the sperm suspension in the proportion of 1:2 and then observed on the optical microscope. Inactive sperm was stained pink, while active sperm was not stained. In total, 500 sperms were counted and the sperm survival rate was calculated.

### 2.3. Ultrastructural Evaluation of Testicles

For this task, two male mice were selected, one from the infected group and the other from the control one. We harvested their testes, and the half of these testes were immersed in 2.5% glutaraldehyde. The testicles were fully filled from one end with the solution. After that, the internal tissue was carefully removed after sliding the blade across albuginea testes, and then divided the size of the tissue into 1 × 1 × 3 mm. The collected samples were fixed overnight in 1% osmic acid, then dehydrated in a series of increasing concentrations of ethanol solution, followed by 100% acetone, and finally, embedded in resin. The ultrathin sections were made and double stained with uranyl acetate and lead citate. After drying, they were observed in transmission electron microscope (TECNAI, Hillsboro, OR, USA). Three parallel samples were prepared.

### 2.4. cDNA Library Construction and Sequencing

Library construction and RNA-Seq were performed according to Illumina’s standard procedures. Firstly, the total RNA was extracted from the preserved testis and uterine samples using the RNAiso Plus (TaKaRa, Dalian, China) according to the manufacturer’s instruction. Then, RNA quality was assessed using a 1.0% agarose gel electrophoresis and a spectrophotometer (Biotek EPOCH, San Francisco, CA, USA). After the initial evaluation, the integrity of the RNA samples was confirmed by the Agilent Bioanalyzer 2100 and RNA 6000 Nano Lab Chip Kit (Agilent Technologies, Santa Clara, CA, USA). Qualified RNA needs to comply with RNA integrity number (RIN) greater than 7.0. To purify the product, poly (A) mRNA was isolated from approximately 10 μg of qualified RNA using poly-T oligo-attached magnetic beads (Invitrogen, Carlsbad, CA, USA). According to the recommendations of the mRNA-Seq sample preparation kit (Illumina, San Diego, CA, USA), these mRNA were divided into small fragments by divalent cations under high temperature. The mRNA fragments were reverse transcribed to create the final cDNA library. Afterward, we performed the paired-end sequencing on an Illumina Hiseq 2000/2500 (Illumina, San Diego, CA, USA) following the vendor’s recommended protocol. 

### 2.5. Sequence Assembly and Acquisition of Clean Reads

The raw data obtained after the previous step were subjected to further filtering to obtain clean reads through removal of the low-quality reads from the raw data. The adaptors in the sequenced reads, which have a ratio of base N ≤ 5% and nucleotides with Q quality score ≥ 20. Q20, Q30, GC%, and the sequence repeatability of clean reads were calculated by Trinity software, as well as the clean reads were stitched. 

### 2.6. Identification and Bioinformatics Analysis of DEGs

The Fragments Per Kilobase of exon model per Million mapped reads (FPKM) was used to calculate the abundance of the gene expression. The FPKM of each gene was calculated by using DEGseq software. In this study, the differently expressed genes (DEGs) of females and males were analyzed separately first, and then DEseq was used to compare the expression of various genes in the testes and uterus. The fold change was calculated based on the normalized gene expression level (FPKM-TOX/FPKM-Control). DEGs were identified as genes with “|log2 (fold change)| ≥ 1, *p*-value < 0.05”. To understand these genes, bioinformatics analysis was applied. At first, the biological functions of the DEGs were enriched through the UniProt database, and then annotated the DEGs by the Gene Ontology (GO) (http://www.geneontology.org/) according to their biological process, molecular function, and cellular component. Finally, the pathway of DEGs was predicted through the Kyoto Encyclopedia of Genes and Genomes (KEGG) (http://www.kegg.jp/kegg/). KEGG facilitates the understanding of the mechanism of different genes’ coordination in the body through formation of an information network. 

### 2.7. Verification of mRNA by Quantitative Real-Time PCR Analysis

After bioinformatics analysis, a series of significant DEGs was obtained. In order to verify whether the expression of these genes in the body is consistent with the results of transcriptome analysis, we performed Quantitative Real-Time PCR Analysis (qRT-PCR). The total RNA was extracted from the preserved testis and uterine samples using RNAiso Plus (TaKaRa, Dalian, China). Concentrations and purity were measured by the spectrophotometer (Biotek EPOCH, San Francisco, CA, USA). Approximately 1 ug total RNA was taken based on the measured concentration, then total RNA was synthesized into cDNA using a reverse transcription kit (Vazyme, Nanjing, China). Gene primers for qRT-PCR were designed by Premier 5.0 software (Table 1). The components were mixed in advance using SYBR qPCR Master Mix (Vazyme, Nanjing, China), and these samples were placed in Roche LifeCycle 96 real-time system at 95 °C for 30 s, followed by 40 cycles of 95 °C for 10 s, and 60 °C for 30 s. Each sample was set to three independent replicates. *β-actin* was applied as an internal standard for normalizing the expression of gene, the blank control group was used as reference sample, which was set to 1. Additionally, relative quantification of target mRNA was performed by using the cycle threshold (Ct) 2^−ΔΔCt^ method [18].

### 2.8. Validations of RNA-Seq Results by Western Blotting

Western blot was used to validate RNA-Seq results, by targeting the protein of two differentially expressed genes, NACHT, LRR, and PYD domains-containing protein 5 (*Nlrp5*) and Interferon-induced protein with tetratricopeptide repeats 3 (*Ifit3*). The pre-processed protein samples were run on SDS-PAGE (80 V at 30 min, 120 V at 120 min), and then transferred into a polyvinylidene fluoride membrane (Millipore, Darmstadt, Germany) for 120 min at 200 mA. The membrane was blocked in 0.1% Tween 20-PBS containing 5% skimmed milk powder for 2 h and then incubated with β-actin mouse monoclonal antibodies (1:10,000 dilution, Proteintech Group, Chicago, USA) and specific mouse anti-*Nlrp5* antibodies (1:1000, Abcam, Massachusetts, USA) for 1 h. Another membrane was incubated with β-actin mouse monoclonal antibodies and anti-*Ifit3* antibodies (1:1000, Abcam, Massachusetts, USA). The membranes were probed with goat anti-mouse IgG conjugated with horseradish peroxidase (HRP) (Tiangen Biotech, Beijing, China) at 1:2500 dilution after washing three times. Finally, the membranes were visualized with BeyoECL Plus (Beyotime, Shanghai, China) and the image was taken and analyzed by the Tanon 5200 Western blotting detection system (Tanon, Shanghai, China).

## 3. Results

### 3.1. Assessment of Reproductive Damage

**Figure 1:**
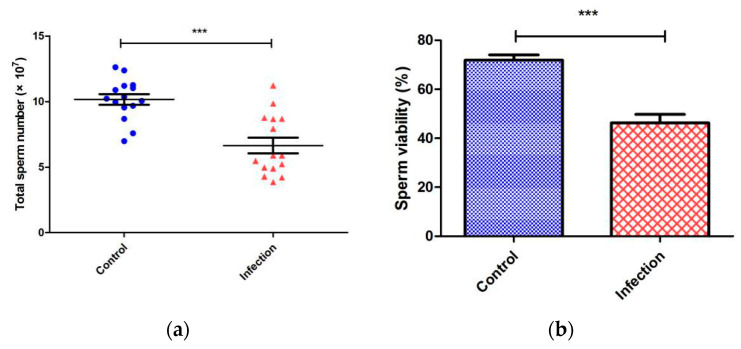
Statistical results of sperm quality parameters during microscopy (*t*-test). (**a**) Scatter plot of total sperm number, control group, and infection group gradually decreased. The whiskers were upper quartile, the median, and lower quartile. The whiskers of the control group were 9.55 × 10^7^, 10.22 × 10^7^, 11.2 × 10^7^. The whiskers of the infection group were 4.89 × 10^7^, 5.88 × 10^7^, 8.68 × 10^7^. (**b**) The histogram of sperm viability indicates that control group and infection group were 71.92% ± 2.11% and 46.28% ± 3.497% (mean ± SEM), respectively. (*** *p* < 0.0001). Error bars represent SEM.

**Figure 2:**
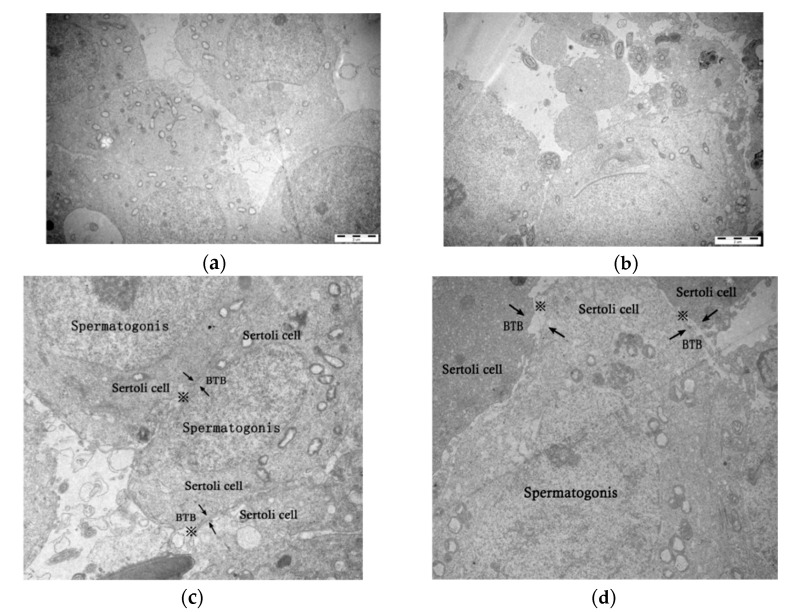
Transmission electron microscope (TEM) micrographs of Kunming (KM) mice testes (6200×). (**a**,**c**) Images belong to the testes of the control mice, (**b**,**d**) are the testes of the acutely infected mice. (**a**) The cell boundaries and the spermatogonia were clearly visible. (**b**) The structure of the testis was destroyed, the cells were incomplete, and the boundary between spermatogonia and Sertoli cells was not obvious. (**c**) The tight junctions (※) between the Sertoli cells were seamless and the blood–testis barrier (BTB) structure was complete. (**d**) The tight junctions’ intercellular spaces (※) between Sertoli cells increase, and the BTB structure was incomplete.

Epididymis is the sperm storage room of mammals. Statistics of the total number of sperms in the epididymis are an important indicator of sperm production efficiency. In addition, calculating the sperm viability can reflect the fertility of male mice. Therefore, we chose the total number of sperm in the epididymis and the sperm survival rate as the sperm quality parameters. The sperm quality parameters of each male mouse in the control group (*n* = 15) and the infected group (*n* = 15) were counted three times and averaged to reduce the error. As a result, the total number of sperms in the control group was in a range of 6.98 ± 0.71 × 10^7^ to 12.62 ± 0.43 × 10^7^, whereas in the infection group, the range was 3.86 ± 0.55 × 10^7^ to 11.22 ± 0.33 × 10^7^ (*p* < 0.05). The average sperm survival rates of the control group and the infected group were 71.92% and 46.28%, respectively, which was also statistically significant, *p* < 0.05 (Figure 1). The ultramarine structure of testes was observed by the transmission electron microscope (TEM) (Figure 2). The testes of mice were damaged after acute infection with *T. gondii*. In control group, ultrathin sections of the testes showed that the Sertoli cells were clearly separated from spermatogonia, and the cells were closely arranged. However, in the infected group, the cell borders were blurred, and the BTB composed of tight junctions between the Sertoli cells was damaged. In addition, the tight junction gaps were significantly increased. This phenomenon is a strong evidence of parasites breaking through the BTB, entering the testes from the blood and causing direct damage to it.

### 3.2. Identification of Expressed Transcripts

**Figure 3:**
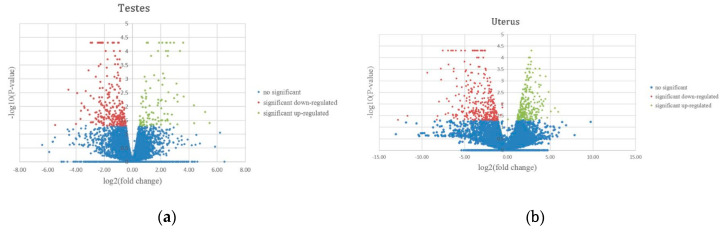
Volcano maps of differentially expressed genes in testes (**a**) and uterus (**b**) compared to control group. Significantly upregulated and downregulated genes are shown as red and green dots, respectively. Blue dots indicate genes that are not significantly different in expression.

**Figure 4:**
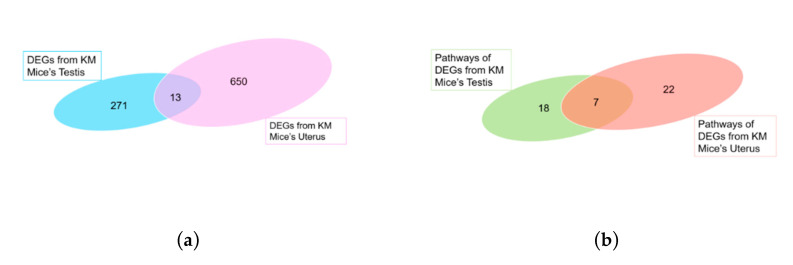
Comparison of transcriptional profiles of testis and uterus during *Toxoplasma gondii* infection. (**a**) Venn diagrams of differently expressed genes (DEGs) between testis and uterus. Genes with base mean |log_2_ (fold change)| ≥ 1, and *p* < 0.05 were collected for analysis. (**b**) Venn diagrams of pathways of DEGs between KM mouse testis and uterus were collected by using the Kyoto Encyclopedia of Genes and Genomes (KEGG) database.

We divided KM mice into four groups for RNA-Seq analysis including two experimental groups and two control groups. These groups formed from male mice infected with *T. gondii* (MaleTox), male mice injected with saline (MaleCtrl), pregnant female mice infected with *T. gondii* (FemaleTox), and pregnant females injected with saline (FemaleCtrl). Three sets of biological replicates are required. Total RNA harvested from the testes and uterus of all mice groups was subjected to high-throughput sequencing on Illumina Hiseq 2000/2500, respectively. The amounts of sequencing data of each sample were more than 6G. In high-throughput sequencing, each measurement of a basic group gives a corresponding value, which is a measure of the accuracy (Table 2). After filtration process, 31,114 (male groups) and 28,718 (female groups) DEGs were obtained. In order to screen the significant DEGs, it is necessary to conform to “*p*-value < 0.05, |log_2_ (fold change)| ≥ 1”. MA-Plots assessed the relationship of log_2_ (FPKM) and log_2_ (fold change) to realize the display of data distribution (see Supplementary file 1). Compared with the control group, the male mice infected group had 291 significantly DEGs, of which 78 and 213 DEGs were labelled as upregulated and downregulated, respectively. On the other hand, the female mice infected group had 679 significantly DEGs, of which 309 and 370 DEGs were labelled as upregulated and downregulated, respectively. The overall distribution of DEGs can be seen by plotting the volcano maps (Figure 3), highlighting the genes with fold change ≥ 1 and *p*-value < 0.05. Among these significant DEGs, we found that 13 genes were co-expressed in the male group and the female group (Figure 4a). 

### 3.3. Gene Ontology of Differentially Expressed Genes

**Figure 5:**
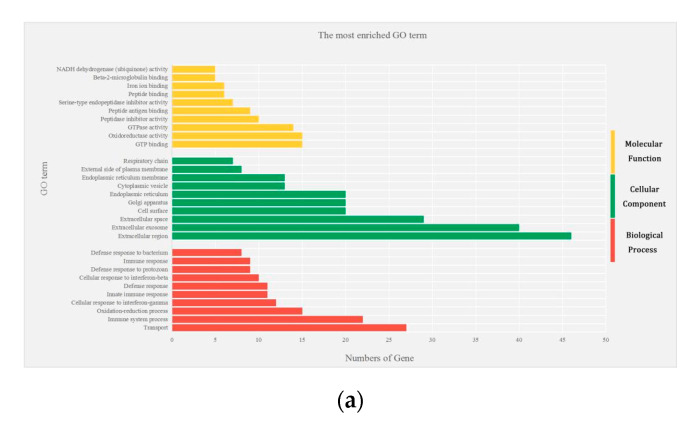

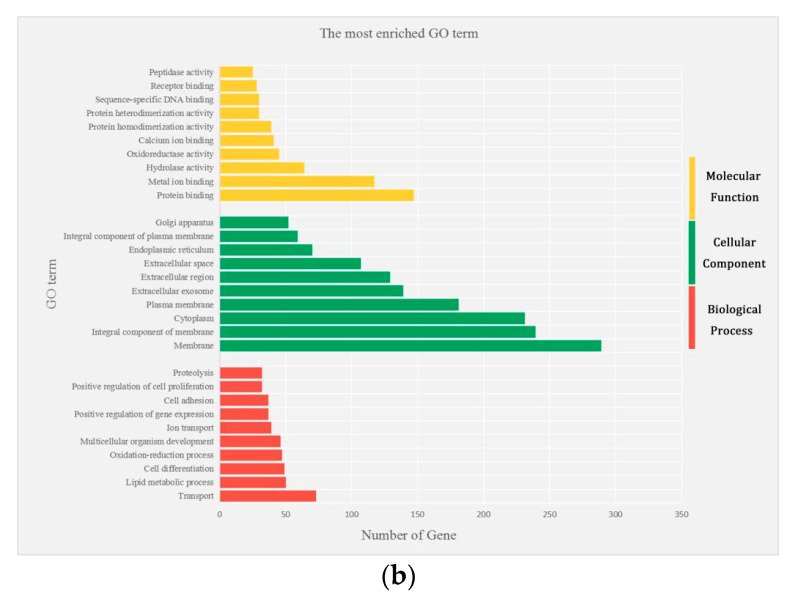
Gene Ontology (GO) distribution of differentially expressed genes of the testes and uterus transcriptome. The top 10 enriched GO terms under biological process, cellular component, and molecular function in (**a**) male mice infected with *T. gondii* and (**b**) female mice infected with *T. gondii*.

The exploration of biological functions of DEGs from other biological processes, cellular components, and molecular functions were carried out by Gene Ontology (GO) enrichment analysis. The distribution diagram was used to describe the top 10 GO terms of each biological function of the male group and female group (Figure 5). In the male group, the main categories of biological processes include transport, immune system process, oxidation-reduction process, and cellular response to interferon. As for cellular components, extra cellular exosome, cell surface, and endoplasmic reticulum are closely related to the cells. Furthermore, in molecular functions, GTP binding, oxidoreductase activity, and GTPase activity occupy a very important part. On the other hand, the female group showed that the biological processes are involved mainly in transport, cell differentiation, and cell adhesion. In respect to the cellular components, most of them were involved in membrane, cytoplasm, and extracellular exosome. Whereas, for molecular functions, it includes protein, calcium ion, and metal ion binding, as well as hydrolase and oxidoreductase activity. Among the GO functions, 35 were involved in both the testes and uterus, representing 11, 9, and 15 involved in biological processes, molecular functions, and cellular components, respectively. The identity of the individual genes within the KEGG pathways and GO terms are shown in Supplementary file 2.

### 3.4. KEGG Enrichment Analysis

**Figure 6:**
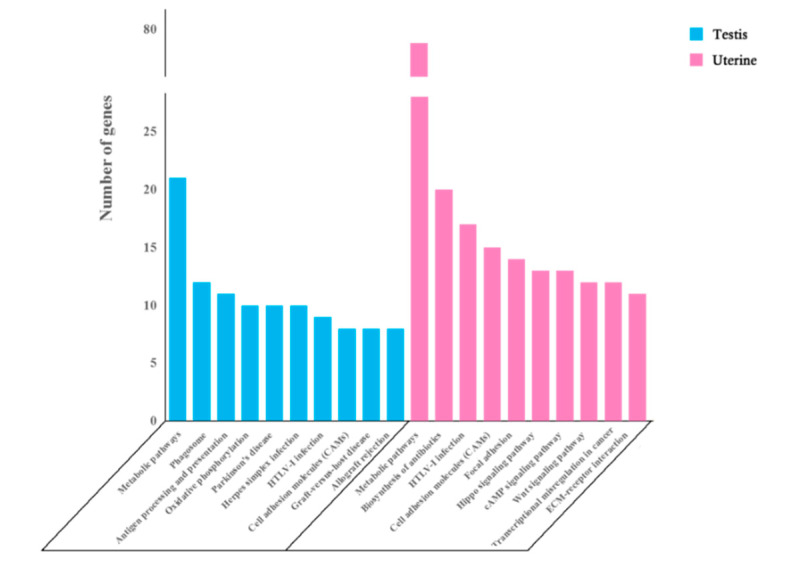
The most enriched pathways of DEGs in testis and uterine during infection. The clustered column shows the top 10 enriched pathways of DEGs from male mice testis and the other 10 of DEGs from the uterus of female mice.

Enrichment analysis of DEGs between the testes and uterus was performed using KEGG database. The male group was significantly involved in 25 pathways, while the female group was involved in 29 pathways. The top 10 enriched pathways in the male group include Metabolic pathways, Phagosome, Antigen processing and presentation, Oxidative phosphorylation, Parkinson’s disease, Herpes simplex infection, HTLV-I infection, Graft-versus-host disease, Cell adhesion molecules (CAMs), and Allograft rejection. Whereas, the top 10 enriched pathways in the female group include Metabolic pathways, Biosynthesis of antibiotics, HTLV-I infection, Cell adhesion molecules (CAMs), Focal adhesion, Hippo signaling pathway, cAMP signaling pathway, Wnt signaling pathway, Transcriptional misregulation in cancer, and ECM–receptor interactions (Figure 6). By comparing these pathways, we found that seven pathways are commonly enriched in male and female groups (Figure 4b), which is briefly described in Table 3.

### 3.5. Verification Results by qRT-PCR Analyzing

**Figure 7:**
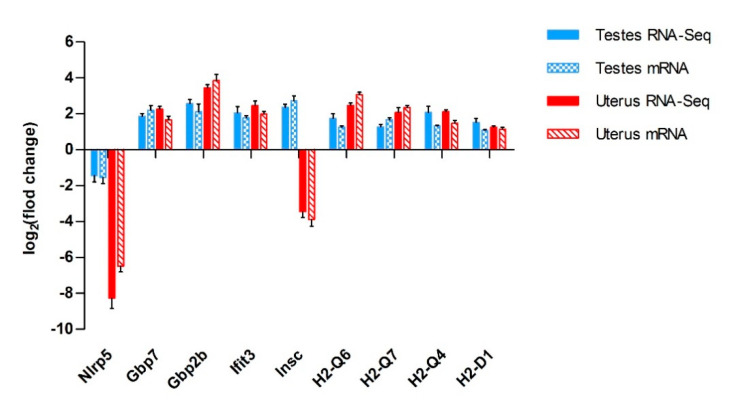
Trend of fold change in mRNA expression, detected by qRT-PCR. The columns show that the nine selected genes verified by qRT-PCR, which have consistent trends with those obtained by RNA-seq. Error bars represent SEM.

Table 4 shows the co-expressed genes and fold changes in the male and female groups. We used qRT-PCR to detect the differential expression trends of these genes to verify the reliability of RNA-Seq results. Through the preliminary analysis of the data, the co-DEGs in the testes and uterus were obtained, and then the relevant literature was searched to find out the genes related to pathogen invasion or barrier function among these co-DEGs. There are some genes, although they are co-DEGs between the testes and uterus, but they were not selected for verification. Here, these results are similar to those of RNA-Seq (Figure 7), and PCR amplification efficiency and primer specificity are shown by amplification curve and melting curve, respectively (see Supplementary file 3).

### 3.6. Verification Results by Western Blotting

**Figure 8:**
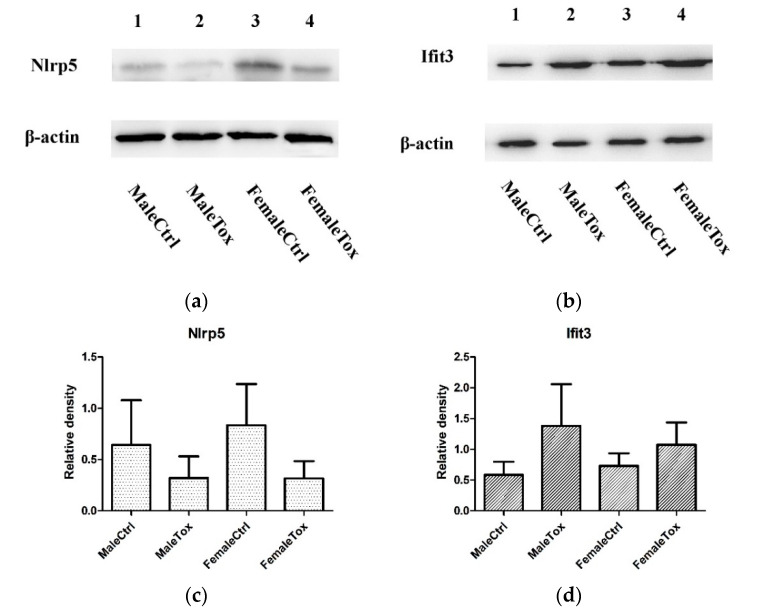
Trend of change in protein expression, detected by Western blotting. The figure shown the change trend of *Nlrp5* and *Ifit3* in testes and uterus during *T. gondii* infection. *β-actin* was used as controls. (**a**,**c**) shown the protein expression and relative density of *Nlrp5* in each group; (**b**,**d**) shown the protein expression and relative density of *Ifit3* in each group; Bar diagram shows changes in the relative density. Error bars represent SEM.

After analyzing the raw data and searching relevant literature, *Nlrp5* (UniprotKB: Q9R1M5, 134 kDa) and *Ifit3* (UniprotKB: Q64345, 56 kDa) were selected as the research targets to explore their role in *T. gondii* infection. Therefore, we verified the expression changes of protein by Western blotting (Figure 8). The results were consistent with the sequencing of the transcriptome, where *T. gondii* infection suppressed *Nlrp5* expression and increased expression of *Ifit3*.

## 4. Discussion

The placental barrier is a special physiological structure that is formed only during the pregnancy of a female mammal. The zygote of mice developed into blastocysts after 3.5 days, and trophoblasts around the blastocyst proliferated close to the endometrium to become syncytial trophoblast cells [19], and the placental barrier began to form. In early pregnancy, the barrier is weak because the placenta is not fully differentiated, and the uterus is tightly integrated with the embryo. From the second trimester, the structure of the placental barrier is fully completed and the barrier function is well developed [20]. In order to achieve a transcriptomic analysis of the effects of *T. gondii* infection on the placental barrier, we chose to infect *T. gondii* in the second trimester of pregnancy (7th day) in mice. Simultaneously, male mice were also infected with *T. gondii* experimentally. In both females and males, we found that *T. gondii* go around the reproductive barrier causing direct damage to the mouse reproductive functions.

Using RNA-seq data analysis, we obtained 28,718 and 31,114 DEGs in FemaleTox group and MaleTox group, respectively. We elaborate the common points of the placental barrier and BTB in case of acute infection with *T. gondii* in respect to DEGs and pathways. In DEGs co-expressed by the uterus and testes, *H2-D1*, *H2-Q4*, *H2-Q6*, and *H2-Q7* were all upregulated. These four genes are the major histocompatibility complex (MHC) located on mouse chromosome 17. The main role of these genes is to present foreign antigens to the body’s immune system, restricting mutual recognition, and inducing immune responses [21]. In particular, the H2 gene is usually upregulated when foreign microorganisms invade the body [22], indicating that *T. gondii* has launched an attack on the mouse’s reproductive system. These four genes are involved in many pathways such as Allograft rejection, Graft-versus-host disease, Autoimmune thyroid disease, HTLV-I infection, and Cell adhesion molecules (CAMs), and may increase the risk of allogeneic rejection, autoimmune disease, and viral infection.

*Nlrp5*, also known as Mater, is a conserved gene mainly expressed in the testes and uterus of adult mammals and related to animal reproductive functions [23]. From our experimental results, we found that *Nlrp5*, as a gene co-expressed by FemaleTox group and MaleTox group, is downregulated compared with the control group, and it is downregulated in the uterus by a factor of more than 304 times (log_2_ (fold change) = 8.46). Tong et al. [24] considered that *Nlrp5* is a crucial gene for early embryonic development. Knock-out of the *Nlrp5* gene in female mice would stagnate the early embryonic development at the two-cell stage, thereby causing infertility. Male mice that lack the *Nlrp5* gene are also infertile. Impaired *Nlrp5* function that causes reduced fertility is ubiquitous in other mammals, such as monkeys [25], bovine [26], and pigs [27]. However, in our experiments, female mice were infected with the parasites in the second trimester of pregnancy, and embryonic development had passed through the two-cell stage. The results showed that *Nlrp5* was still significantly downregulated. The reason may be that *Nlrp5* can not only affect the early embryonic development, but also the subsequent fetal development. Interestingly, with the aging of animals, the content of *Nlrp5* in the oocytes will also decrease, indicating that the decline in the fertility of older females may be related to the decrease or loss of *Nlrp5* [28]. Moreover, some studies have pointed out that *Nlrp5* mediates the mitochondrial function in mouse embryos, thus, the lack of *Nlrp5* in the mother’s body will automatically activate the mitochondrial pool prematurely leading to embryonic mitochondrial damage [29]. It is expected that *Nlrp5* may have more complex functions during the maternal pregnancy. Although *Nlrp5* is expressed in the testes, this seems to be not related to the reports showing the function of this gene in male animals. Up to now, studies are focusing mainly on the effect of *Nlrp5* on the maternal pregnancy. Our experimental results showed that as a pathogen, the invasion process of *T. gondii* results in downregulation of *Nlrp5*, especially in the female mice. In this study, we tested the fertility of mice and the results showed that *T. gondii* can also reduce the fertility of male mice. At present, there is little report on the relationship between *T. gondii* and *Nlrp5*. According to the results of the current study, *Nlrp5* may be a new target for studying the reproductive damage caused by *T. gondii*, which breaking through the reproductive system barrier.

*Insc* is a weight that controls the balance between symmetric cell division (SCD) and asymmetric cell division (ACD). Tissue homeostasis requires an ordered cell division. SCD enhances the proliferation of cells, meanwhile, ACD promotes the tissue differentiation and ensures the cell diversity [30]. In our present study, *Insc* showed a very different expression in the two different reproductive systems after the invasion of *T. gondii*. The gene is upregulated five times, (log_2_ (flod change) = 2.35) in the MaleTox group and downregulated 11 times (log_2_(flod change) = −3.46) in the FemaleTox group (Table 3). Since *Insc* interacts with Par3 and LGN, and participates in ACD, *Insc* has long been considered as the molecular connection between the apically localized complex (Par3/Par6/aPKC) and the microtubule-associated complex (NuMA/LGN/Gai). It promotes spindle-polarity coupling during the mitosis process leading to an unequal cell division [31,32]. Recent studies have found that high expression of *Insc* will drive the dividing mode of cells to SCD, resulting in an increase in the cell number and volume [33]. In both cases of the upregulation or downregulation of *Insc*, it may cause the tissue to lose its differentiation function but it can only increase the surface area. It is suggested that the change in the expression of *Insc* may lead to several pathological reactions in the host and adverse maternal pregnancy. After infection with *T. gondii*, the opposite change in the expression of *Insc* in male and female mice may be explained by the different mechanisms of *T. gondii* infection due to the wide difference in the male and female reproductive systems. It is recommended to combine immunological and pathological experiments for further investigations.

Interferon-inducible Guanylate-binding protein (*Gbp*, 65–72 kDa) can protect organisms from being infected and facilitate the proinflammatory signal transduction by promoting the host cells’ death [34]. There are seven types of *Gbps* reported in human genome (*hGbp1* to *hGbp7*), whereas 11 types of *Gbps* in murine genome (*mGbp 1–11*). During infection of exogenous pathogenic microorganism, many types of murine *Gbps* [35,36,37] and human *Gbp2* and *Gbp5* [38,39] can trigger the host cells’ pyroptosis by activating inflammasome complex containing caspase-1 or caspase-4. After infection of *T. gondii*, *mGbp2B* (also named *Gbp1*) can activate inflammasome complex signaling cascades, which leads to *Nlrp1*, *Nlrp3*, *Nlrp4*, and pro-caspase-1 degradation resulting in converting the cell death type from pyroptosis to apoptosis [40]. Mouse guanylate-binding protein7 (*mGbp7*) exhibits high affinity for GTP, nevertheless; it is inferior to the members of small GTPase families such as Ras and Gα. Additionally, it is one of the members with highest affinity in *mGbp* counterparts [41,42], which enables *mGbp7* to hydrolyze GTP more efficiently to promote the host defense capability to some extent. In addition, the vimineous C-terminal tail of *mGbp7*, composed of 49 amino acid residues, can play a similar role to the C-terminal CaaX motif of other *mGbps* to function as anchorage by being inserted into intramembrane [42]. Although *mGbp7* and other *mGbps* (*mGbp1*, *-2*, *-3*, *-6*, and *-9*) can effectively recruit *T. gondii* [43], the CT tail on *mGbp7* hardly migrates into the parasites’ parasitophorous vacuole [42]. This interesting phenomenon suggests that there may be an unknown mechanism of CT tail, which plays an important role in membrane protein localization, membrane anchoring, or interaction.

The interferon-induced proteins with tetratricopeptide repeats (*Ifits*) are a conserved family of antiviral RNA-binding proteins in vertebrates, and play a significant role in antiviral immune responses [44]. On the one hand, *Ifit3*, as an intermediate molecule, stabilizes the normal expression of *Ifit1* in the host cells helping them to recognize the virus invasion and make a rapid inhibitory response [45]. On the other hand, the tetrapeptide repeat motif of *Ifit3* interacts with the N terminus of TBK1 making a bridging protein that connects TBK1 to MAVS on the mitochondria, and is important for protein transport, translational initiation, cell migration and proliferation, antiviral signaling, and virus replication. Recent studies have found that the protection mechanism of *Ifit3* for the host is not only against viruses, but is also effective against tumors such as breast cancer [46], and oral squamous cell carcinoma [47], as well as bacteria and parasite infections such as pneumococcus [48], plasmodium [49]. Among the immune proteins induced by Interferon gamma (IFN-γ), *Gbps* is one of the most abundant proteins. IFN-γ plays a vital role in antimicrobial defense. The lack of IFN-γ receptor signaling in the mammals results in loss of function or mutation of IFN-γ receptor signaling, which can significantly reduce the defense ability of invading foreign microorganisms and make the host extremely susceptible to *T. gondii* [50]. *T. gondii* can induce the death of infected cells and promote its own transmission in the host. This situation includes the mechanism of apoptosis. The inhibition of apoptosis varies depending on the type of host and the presence of IFN-γ. *Ifits* and *Gbps* proteins belong to IFN-α and IFN-γ stimulated genes (ISGs). Interestingly, researchers are accustomed to using *Ifit3* and *Gbps* as a marker to distinguish between type I interferons (IFN-α and IFN-β) and type II interferons (IFN-γ) [51]. The upregulated expression of marker proteins just shows that the reproductive system can initiate the defense effect of interferon to resist the invasion of *T. gondii*. *Gbps* is an interferon-induced protein that can resist the attack of pathogens. Several reports demonstrated the shape of co-expression relationship between *Gbps* and *Ifit3* [52,53,54]. Philippe et al. suggested that ISGs may induce different proteins to cooperate in the control of the production of specific type of IFN [55]. The current mainstream view revealed that the host’s autoimmune defense is induced by IFN-γ after *T. gondii* infection [56]. Type I interferon is mainly involved in the immune system regulation and control of antiviral factors to prevent viral infection. INF-α induces NK cells to produce IFN-γ [57], which is an upstream event of IFN-γ against parasitic infection. The upregulation of *Ifit3* may be essential to promote the IFN-α induced expression of IFN-γ in the NK cells, and ultimately enhances the host’s functional ability to resist the parasitic infection. On the other hand, research studies investigating the relationship between *Ifit3* and *T. gondii* is scanty. The research on the relationship between *Ifit3* and parasites may help us to understand the defense mechanism of the interferon of the host against *T. gondii* infection.

## 5. Conclusions

Results of this study confirm that *T. gondii* can result in serious reproductive damage in both genders such as decreased sperm count, decreased sperm survival rate, and poor pregnancy in pregnant mice. We also provided transcriptome data of differentially expressed genes in testes and uterus after *T. gondii* infection and provided data to support subsequent research on parasite invasion to the reproductive system. From the investigated data, we screened a series of genes that can respond to *T. gondii* infection in the reproductive system of both sexes. They may be the key members to lend a hand to *T. gondii* to cross through the tissue barrier and cause the subsequent damage to the tissues.

## Figures and Tables

**Table 1:** Design of primers used for Quantitative Real-Time PCR Analysis (qRT-PCR) analysis.

Gene ID	Gene	Forward Primer	Reverse Primer	Product Length
23968	*Nlrp5*	ACTGGGAAAGAGATGCACCC	GCCATCATGCACAAACCCAC	147
229900	*Gbp7*	AGCTCATTGGGTGCAAAAATCC	ACTTACCAGAACCAGGCACTAC	73
14468	*Gbp2b*	GCCTCGCCTGTGTATCAGGA	GGAAAGTTGCTTCCTGTCTCC	82
15959	*Ifit3*	TCCTCCAAAAGGCAGCTCAG	TCCCTGTAACGGCACATGAC	133
233752	*Insc*	GCAGGTAGACTCGGTTCAGC	AGGTCACCTTGCGTGTCTTC	117
110557	*H2-Q6*	CCCCAGGTCTTATGGTGCTG	TGCCAAGTCAGGGTGATGTC	78
15018	*H2-Q7*	CCCAGGTCTTATGGTGCTGTC	TCAGCTCCTCCCCATTCAAC	97
15015	*H2-Q4*	GAAAGCCAAGGGCAATGAGC	CCGCGCTCTGGTTGTAGTAG	74
14964	*H2-D1*	GACAACAAGGAGTTCGTGCG	CACTCGGAACCACTGCTCTT	144
11461	*β-actin*	AGAGAAGCTGTGCTATGTTGCT	GGAACCGCTCGTTGCCAATA	128

**Table 2:** Pretreatment results of reads data in the high throughput sequencing.

Sample	Raw Data		Valid Data		Valid Ratio (Reads)	Q20%	Q30%	GC Content%
	Read	Base	Read	Base				
MaleCtrl-1	51722480	7.76 G	51153928	7.67 G	98.9	99.68	96.19	50
MaleTox-1	53513324	8.03 G	52647430	7.90 G	98.38	99.65	96.94	50
FemaleCtrl-1	49853240	7.48 G	48944858	7.34 G	98.18	99.75	97.42	49.5
FemaleTox-1	44228168	6.63 G	43353818	6.50 G	98.02	99.63	96.99	50
MaleCtrl-2	50455024	7.57 G	49652644	7.45 G	98.41	99.64	96.95	50.5
MaleTox-2	59110516	8.87 G	58525676	8.78 G	99.01	99.73	97.16	50.5
FemaleCtrl-2	53600952	7.33 G	52597978	7.18 G	98.13	98.68	96.15	51
FemaleTox-2	43890326	6.58 G	42897454	6.44 G	97.74	98.93	93.25	49.5
MaleCtrl-3	48768472	7.32 G	48078448	7.21 G	98.59	99.69	96.9	49
MaleTox-3	54269660	8.14 G	53547754	8.03 G	98.67	99.69	97.05	49.5
FemaleCtrl-3	52033912	7.81 G	51570014	7.74 G	99.11	99.65	96.78	50.5
FemaleTox-3	44641116	6.70 G	43937778	6.59 G	98.42	99.69	96.97	50

**Table 3:** Common pathway in the testes and uterus of infected mice.

KEGG ID	Pathway	Description	Gene Numbers in Testes	Gene Numbers in Uterus
mmu05320	Autoimmune thyroid disease	Includes Graves’ disease (GD) and autoimmune atrophic thyroiditis or primary myxedema, Hashimoto’s thyroiditis (HT) or chronic autoimmune thyroiditis and its variants.	8	7
mmu05166	HTLV-I infection	A retrovirus, involved in many naturally occurring neoplasms in various animal species.	9	17
mmu05330	Allograft rejection	The rejection of a donor tissue graft by the immune system of a recipient.	8	7
mmu05332	Graft-versus-host disease	An immunological disorder that affects many organ systems by allografts.	8	6
mmu01100	Metabolic pathways	Metabolic process consisting of a series of biochemical reactions, such as glycolytic pathway, tricarboxylic acid cycle pathway, gluconeogenesis pathway, fatty acid synthesis pathway.	21	79
mmu04940	Type I diabetes mellitus	An autoimmune disease of endocrine system characterized by minimal or absent production of insulin by the pancreas.	8	7
mmu04514	Cell adhesion molecules (CAMs)	A group of membrane glycoprotein and carbohydrate molecules that mediate the adhesion of cells to cells or of cells to the extracellular matrix.	8	15

**Table 4:** Genes in the testes and uterus of infected mice.

Gene Symbol	Fold Change in Testes	Fold Change in Uterus	Description
*Kcnk2*	−3.528649146	−24.02119933	A two-pore domain potassium channel involved in maintaining cellular membrane resting potentials.
*Nlrp5*	−2.342730135	−304.5906692	Closely related to early embryo development.
*Gbp7*	3.563261125	4.890800334	A GBP family member with an unusual and extended C-terminus.
*Gbp2b*	−4.040695723	−7.340503123	Gbp2b plays an important role in activating inflammatory bodies and mediating resistance to intracellular pathogens.
*Ceacam10*	5.871390465	10.7128078	Ceacam10 is a kind of glycoprotein in mouse seminal vesicle secretions that enhances sperm motility and is essential during placental and embryonic development.
*Kcnq2*	−4.833063041	15.11249898	KCNQ2 combines with KCNQ3 to form a potassium ion channel, it is important in the regulation of neuronal excitability.
*Pappa2*	−15.07331338	−13.54553907	Affects fetal bone development. Decreased expression of Pappa2 will affect the body size of the fetus at birth.
*Ifit3*	4.073777097	5.433739017	It plays a vital role in the resistance of interferon-α to pathogens.
*Insc*	5.101441093	−11.00866683	Induced spindles to perform unequal mitosis on cells and promote cell differentiation.
*H2-Q6*	3.454653679	5.466215554	The H2 system determines which components of the cell membrane can induce cellular and humoral responses with proper donor-acceptor binding. They are of great significance in regulating innate and adaptive immune responses and are closely related to many infectious and/or autoimmune diseases.
*H2-Q7*	2.322132722	4.201589866	
*H2-D1*	2.911529549	2.438249553	
*H2-Q4*	4.134029458	4.313827783	

## Data Availability

Raw data has been published on the Sequence Read Archive (SRA). Accession ID: PRJNA632742. Processed data of the RNA-Seq analysis is shown in Supplementary file 4.

## Supplementary Materials

The following are available online at https://www.mdpi.com/2076-2607/8/8/1136/s1.

## References

[1] Jones J.L., Dubey J.P. (2012). Foodborne toxoplasmosis. Clin. Infect. Dis..

[2] Smadja D., Cabre P., Prat C., Vernant J.C. (1995). Loss of psychic auto-activation. Obsessive-compulsive behavior. Toxoplasmic abscess of the basal ganglia. Rev. Neurol..

[3] Johnson S.K., Fitza M.A., Lerner D.A., Calhoun D.M., Beldon M.A., Chan E.T., Johnson P.T.J. (2018). Risky business: Linking Toxoplasma gondii infection and entrepreneurship behaviours across individuals and countries. Proc. Biol. Sci..

[4] Hill D.E., Chirukandoth S., Dubey J.P. (2005). Biology and epidemiology of Toxoplasma gondii in man and animals. Anim. Health Res. Rev..

[5] Saadatnia G., Golkar M. (2012). A review on human toxoplasmosis. Scand. J. Infect. Dis..

[6] Barreto F., Hering F., Dall’oglio M.F., Martini filho D., Campagnari J.C., Srougi M. (2008). Toxoplasmosis testicular: Un caso raro de masa testicular. Actas Urol. Esp..

[7] Colosi H.A., Jalali-Zadeh B., Colosi I.A., Simon L.M., Costache C.A. (2015). Influence ofToxoplasma gondiiInfection on Male Fertility: A Pilot Study on Immunocompetent Human Volunteers. Iran. J. Parasitol..

[8] Lopes W.D.Z., Costa A.J., Souza F.A., Rodrigues J.D.F., Costa G.H.N., Soares V.E., Silva G.S. (2009). Semen variables of sheep (Ovis aries) experimentally infected with Toxoplasma gondii. Anim. Reprod. Sci..

[9] Teixeira W.F.P., Tozato M.E.G., Pierucci J.C., Vital G.P., Cruz A.C., Lopes W.D.Z., Cursino M.S., Joaquim S.F., Soares V.E., Langoni H. (2017). Investigation of Toxoplasma gondii in semen, testicle and epididymis tissues of primo-infected cats (Felis catus). Vet. Parasitol..

[10] Bajic G., Degn S.E., Thiel S., Andersen G.R. (2015). Complement activation, regulation, and molecular basis for complement-related diseases. Embo J..

[11] Crider S.R., Horstman W.G., Massey G.S. (1988). Toxoplasma orchitis: Report of a case and a review of the literature. Am. J. Med..

[12] Pietroiusti A., Campagnolo L., Fadeel B. (2013). Interactions of engineered nanoparticles with organs protected by internal biological barriers. Small.

[13] Blundell C., Tess E.R., Schanzer A.S., Coutifaris C., Su E.J., Parry S., Huh D. (2016). A microphysiological model of the human placental barrier. Lab. Chip..

[14] Huang W.Y., Wang Y.P., Mahmmod Y.S., Wang J.J., Liu T.H., Zheng Y.X., Zhou X., Zhang X.X., Yuan Z.G. (2019). A Double-Edged Sword: Complement Component 3 in Toxoplasma gondii Infection. Proteomics.

[15] Lockhart D.J., Winzeler E.A. (2000). Genomics, gene expression and DNA arrays. Nature.

[16] Wang Z., Gerstein M., Snyder M. (2009). RNA-Seq: A revolutionary tool for transcriptomics. Nat. Rev. Genet..

[17] Li X.Z., Wang X.H., Xia L.J., Weng Y.B., Hernandez J.A., Tu L.Q., Li L.T., Li S.J., Yuan Z.G. (2015). Protective efficacy of recombinant canine adenovirus type-2 expressing TgROP18 (CAV-2-ROP18) against acute and chronic Toxoplasma gondii infection in mice. BMC Infect. Dis..

[18] Livak K.J., Schmittgen T.D. (2001). Analysis of relative gene expression data using real-time quantitative PCR and the 2(-Delta Delta C(T)) Method. Methods.

[19] Newby D., Marks L., Cousins F., Duffie E., Lyall F. (2005). Villous Explant Culture: Characterization and Evaluation of a Model to Study Trophoblast Invasion. Hypertens. Pregnancy.

[20] Robbins J.R., Zeldovich V.B., Poukchanski A., Boothroyd J.C., Bakardjiev A.I. (2012). Tissue barriers of the human placenta to infection with Toxoplasma gondii. Infect. Immun..

[21] Shiina T., Blancher A., Inoko H., Kulski J.K. (2017). Comparative genomics of the human, macaque and mouse major histocompatibility complex. Immunology.

[22] Leroux L.P., Nishi M., El-Hage S., Fox B.A., Bzik D.J., Dzierszinski F.S. (2015). Parasite Manipulation of the Invariant Chain and the Peptide Editor H2-DM Affects Major Histocompatibility Complex Class II Antigen Presentation during Toxoplasma gondii Infection. Infect. Immun..

[23] Tong Z.B., Nelson L.M., Dean J. (2000). Materencodes a maternal protein in mice with a leucine-rich repeat domain homologous to porcine ribonuclease inhibitor. Mamm. Genome.

[24] Tong Z.B., Lyn G., Anto D.P., Konstantina V., Heidi D., Paola S., Carla P., Bondy C.A., Nelson L.M. (2004). Developmental Expression and Subcellular Localization of Mouse MATER, an Oocyte-Specific Protein Essential for Early Development. Endocrinology.

[25] Wu X. (2009). Maternal depletion of NLRP5 blocks early embryogenesis in rhesus macaque monkeys (Macaca mulatta). Hum. Reprod..

[26] Pennetier S., Perreau C., Uzbekova S., Thelie A., Delaleu B., Mermillod P., Dalbies-Tran R. (2006). MATER protein expression and intracellular localization throughout folliculogenesis and preimplantation embryo development in the bovine. BMC Dev. Biol..

[27] Ma J., Milan D., Rocha D. (2009). Chromosomal assignment of the porcine NALP5 gene, a candidate gene for female reproductive traits. Anim. Reprod. Sci..

[28] Lu Y.Q., He X.C., Zheng P. (2016). Decrease in expression of maternal effect gene Mater is associated with maternal ageing in mice. Mol. Hum. Reprod..

[29] Fernandes R., Tsuda C., Perumalsamy A.L., Naranian T., Chong J., Acton B.M., Tong Z.B., Nelson L.M., Jurisicova A. (2012). NLRP5 mediates mitochondrial function in mouse oocytes and embryos. Biol. Reprod..

[30] Poulson N.D., Lechler T. (2010). Robust control of mitotic spindle orientation in the developing epidermis. J. Cell Biol..

[31] Bultje R.S., Castaneda-Castellanos D.R., Jan L.Y., Jan Y.N., Kriegstein A.R., Shi S.H. (2009). Mammalian Par3 regulates progenitor cell asymmetric division via notch signaling in the developing neocortex. Neuron.

[32] Williams S.E., Fuchs E. (2013). Oriented divisions, fate decisions. Curr. Opin. Cell Biol..

[33] Ballard M.S., Zhu A., Iwai N., Stensrud M., Mapps A., Postiglione M.P., Knoblich J.A., Hinck L. (2015). Mammary Stem Cell Self-Renewal Is Regulated by Slit2/Robo1 Signaling through SNAI1 and mINSC. Cell Rep..

[34] Meunier E., Dick M.S., Dreier R.F., Schurmann N., Kenzelmann Broz D., Warming S., Roose-Girma M., Bumann D., Kayagaki N., Takeda K. (2014). Caspase-11 activation requires lysis of pathogen-containing vacuoles by IFN-induced GTPases. Nature.

[35] Finethy R., Luoma S., Orench-Rivera N., Feeley E.M., Haldar A.K., Yamamoto M., Kanneganti T.-D., Kuehn M.J., Coers J.r., Weiss D.S. (2015). Inflammasome Activation by Bacterial Outer Membrane Vesicles Requires Guanylate Binding Proteins. mBio.

[36] Meunier E., Broz P. (2016). Interferon-inducible GTPases in cell autonomous and innate immunity. Cell. Microbiol..

[37] Meunier E., Wallet P., Dreier R.F., Costanzo S., Anton L., Ruhl S., Dussurgey S., Dick M.S., Kistner A., Rigard M. (2015). Guanylate-binding proteins promote activation of the AIM2 inflammasome during infection with Francisella novicida. Nat. Immunol..

[38] Britzen-Laurent N., Bauer M., Berton V., Fischer N., Syguda A., Reipschlager S., Naschberger E., Herrmann C., Sturzl M. (2010). Intracellular trafficking of guanylate-binding proteins is regulated by heterodimerization in a hierarchical manner. PLoS ONE.

[39] Shenoy A.R., Wellington D.A., Kumar P., Kassa H., Booth C.J., Cresswell P., Macmicking J.D. (2012). GBP5 Promotes NLRP3 Inflammasome Assembly and Immunity in Mammals. Science.

[40] Fisch D., Bando H., Clough B., Hornung V., Yamamoto M., Shenoy A.R., Frickel E.M. (2019). Human GBP1 is a microbe-specific gatekeeper of macrophage apoptosis and pyroptosis. Embo J..

[41] Legewie L., Loschwitz J., Steffens N., Prescher M., Wang X., Smits S.H.J., Schmitt L., Strodel B., Degrandi D., Pfeffer K. (2019). Biochemical and structural characterization of murine GBP7, a guanylate binding protein with an elongated C-terminal tail. Biochem. J..

[42] Praefcke G.J.K., Geyer M., Schwemmle M., Kalbitzer H.R., Herrmann C. (1999). Nucleotide-binding characteristics of human guanylate-binding protein 1 (hGBP1) and identification of the third GTP-binding motif. J. Mol. Biol..

[43] Degrandi D., Konermann C., Beuter-Gunia C., Kresse A., Würthner J., Kurig S., Beer S., Pfeffer K. (2007). Extensive characterization of IFN-induced GTPases mGBP1 to mGBP10 involved in host defense. J. Immunol..

[44] Mears H.V., Sweeney T.R. (2018). Better together: The role of IFIT protein-protein interactions in the antiviral response. J. Gen. Virol..

[45] Johnson B., VanBlargan L.A., Xu W., White J.P., Shan C., Shi P.Y., Zhang R., Adhikari J., Gross M.L., Leung D.W. (2018). Human IFIT3 Modulates IFIT1 RNA Binding Specificity and Protein Stability. Immunity.

[46] Legrier M.E., Bieche I., Gaston J., Beurdeley A., Yvonnet V., Deas O., Thuleau A., Chateau-Joubert S., Servely J.L., Vacher S. (2016). Activation of IFN/STAT1 signalling predicts response to chemotherapy in oestrogen receptor-negative breast cancer. Br. J. Cancer.

[47] Pidugu V.K., Wu M.M., Yen A.H., Pidugu H.B., Chang K.W., Liu C.J., Lee T.C. (2019). IFIT1 and IFIT3 promote oral squamous cell carcinoma metastasis and contribute to the anti-tumor effect of gefitinib via enhancing p-EGFR recycling. Oncogene.

[48] Skovbjerg S., Norden R., Martner A., Samuelsson E., Hynsjo L., Wold A.E. (2017). Intact Pneumococci Trigger Transcription of Interferon-Related Genes in Human Monocytes, while Fragmented, Autolyzed Bacteria Subvert This Response. Infect. Immun..

[49] Liehl P., Meireles P., Albuquerque I.S., Pinkevych M., Baptista F., Mota M.M., Davenport M.P., Prudencio M. (2015). Innate immunity induced by Plasmodium liver infection inhibits malaria reinfections. Infect. Immun..

[50] Janssen R., Van Wengen A., Verhard E., De Boer T., Zomerdijk T., Ottenhoff T.H., Van Dissel J.T. (2002). Divergent role for TNF-alpha in IFN-gamma-induced killing of Toxoplasma gondii and Salmonella typhimurium contributes to selective susceptibility of patients with partial IFN-gamma receptor 1 deficiency. J. Immunol..

[51] Hall J.C., Casciola-Rosen L., Berger A.E., Kapsogeorgou E.K., Cheadle C., Tzioufas A.G., Baer A.N., Rosen A. (2012). Precise probes of type II interferon activity define the origin of interferon signatures in target tissues in rheumatic diseases. Proc. Natl. Acad. Sci. USA.

[52] Lacaze P., Raza S., Sing G., Page D., Forster T., Storm P., Craigon M., Awad T., Ghazal P., Freeman T.C. (2009). Combined genome-wide expression profiling and targeted RNA interference in primary mouse macrophages reveals perturbation of transcriptional networks associated with interferon signalling. BMC Genom..

[53] Moody L.R., Herbst A.J., Aiken J.M. (2011). Upregulation of interferon-gamma-induced genes during prion infection. J. Toxicol. Environ. Health A.

[54] Zhang S., Kim C.C., Batra S., McKerrow J.H., Loke P. (2010). Delineation of diverse macrophage activation programs in response to intracellular parasites and cytokines. PLoS Negl. Trop. Dis..

[55] Metz P., Dazert E., Ruggieri A., Mazur J., Kaderali L., Kaul A., Zeuge U., Windisch M.P., Trippler M., Lohmann V. (2012). Identification of type I and type II interferon-induced effectors controlling hepatitis C virus replication. Hepatology.

[56] Sasai M., Pradipta A., Yamamoto M. (2018). Host immune responses to Toxoplasma gondii. Int. Immunol..

[57] Koblansky A.A., Jankovic D., Oh H., Hieny S., Sungnak W., Mathur R., Hayden M.S., Akira S., Sher A., Ghosh S. (2013). Recognition of profilin by Toll-like receptor 12 is critical for host resistance to Toxoplasma gondii. Immunity.

