# Supplementary material for: Transcriptome Analysis of Testes and Uterus: Reproductive Dysfunction Induced by Toxoplasma gondii in Mice
Source: Microorganisms. 2020 Jul 28;8(8):1136. doi: 10.3390/microorganisms8081136 (PMC7464677; doi:10.3390/microorganisms8081136)
Supplement: Supplementary file 1 [file microorganisms-08-01136-s001.zip › Supplementary file/20200703Supplementary file 3.doc]

（a）

（b）

S3.Figure 1. The amplification curve and melting curve of primer Gbp2b

（a）

（b）

S3.Figure 2. The amplification curve and melting curve of primer Gbp7

（a）

（b）

S3.Figure 3. The amplification curve and melting curve of primer Ifit3

（a）

（b）

S3.Figure 4. The amplification curve and melting curve of primer Nlrp5

（a）

（b）

S3.Figure 5. The amplification curve and melting curve of primer H2-Q7

（a）

（b）

S3.Figure 6. The amplification curve and melting curve of primer Insc

（a）

（b）

S3.Figure 7. The amplification curve and melting curve of primer H2-Q6

（a）

（b）

S3.Figure 8. The amplification curve and melting curve of primer H2-D1

（a）

（b）

S3.Figure 9. The amplification curve and melting curve of primer H2-Q4
